# MicroRNA expression profiling after recurrent febrile seizures in rat and emerging role of miR-148a-3p/SYNJ1 axis

**DOI:** 10.1038/s41598-020-79543-0

**Published:** 2021-01-13

**Authors:** Jian Xu, Mingqiang Sun, Xiaodong Li, Lei Huang, Zhenzhong Gao, Jian Gao, Anmu Xie

**Affiliations:** 1 Department of Neurology, Maternal and Child Health Hospital of Weifang Medical University, Weifang, 261011 China; 2 Department of Clinical Lab, Maternal and Child Health Hospital of Weifang Medical University, Weifang, 261011 China; 3 Department of Pediatric, Maternal and Child Health Hospital of Weifang Medical University, Weifang, 261011 China; 4 Department of Cancer Blood Disease, Cincinnati Children’s Hospital Medical Center, Cincinnati, OH 45229 USA

**Keywords:** Cell death, Mechanisms of disease, Diseases, Neurology, Neurological disorders

## Abstract

Febrile seizures (FSs) are common neurological disorders in both infants and children, although the precise underlying mechanism remains to be explored, especially in the expression pattern and function of microRNAs (miRNAs). In this report, we aimed to screen new potential miRNAs and examine the role of miR-148a-3p in hippocampal neurons in FS rats via Synaptojanin-1 (SYNJ1). Thirty rats were randomly divided into the normal and FS model groups, which were investigated by miRNA array. This process identified 31 differentially expressed (20 upregulated and 11 downregulated) miRNAs and potential miRNA target genes. In addition, hippocampal neurons were assigned into five groups for different transfections. Apoptosis was detected by TUNEL and flow cytometry. SYNJ1 was identified as a target gene of miR-148-3p. In vitro experiments revealed that inhibition of miR-148a-3p decreased neuronal cell apoptosis. Moreover, overexpression of miR-148a-3p resulted in activation of PI3K/Akt signaling pathway and the apoptosis of hippocampal neurons. MiR-148a-3p inhibitor could reverse the above events. Taken together, our data demonstrated that the hippocampal miRNA expression profiles of a rat model of FS provide a large database of candidate miRNAs and neuron-related target genes. Furthermore, miR-148a-3p acted as a apoptosis enhcaner via the activation of the SYNJ1/PI3K/Akt signaling pathway, highlighting a potential therapeutic target in the treatment of infants with hyperthermia-induced brain injury.

## Introduction

Febrile seizures (FSs) are common in both infants and children, and they occur in approximately 2% of the child population and are the most common neurological disease in children^1^. According to the American Academy of Pediatrics (AAP), FSs can occur in the absence of metabolic disorders, intracranial infections, or a history of seizures and can be classified as simple or complex FSs^2,3^. These types of FSs are associated with temperatures of 39 °C or higher, although there is a lack of evidence for specific causes, such as acute electrolyte imbalances^4^. Developmental delays, viral infections, discharge from the neonatal unit after 28 days, a family history of febrile spasms, and possible iron and zinc deficiencies have been identified as risk factors for FSs^5^. Antipyretic therapy alone is ineffective; however, this therapy is effective in combination with phenobarbital in preventing the recurrence of FSs and is even more effective in combination with phenobarbital and valproic acid in routine antiepileptic therapy^6^. Due to the undesirable side effects of these methods, new biomarkers are urgently needed to improve FS diagnosis, treatment and prognosis.

In recent years, the in-depth study of noncoding RNAs has prompted the roles of small molecular RNAs in neurological diseases, and especially the relationships between small molecular RNAs and brain development, to receive increasing attention^7^. MicroRNAs (miRNAs) compose a family of noncoding small RNAs and are essential posttranscriptional regulators that inhibit mRNA translation or directly degrade target mRNA^8^. They are essential for the healthy development of neurons and may be involved in many neurological diseases; however, the mechanisms of such involvement remain to be explored. For example, miRNAs play essential roles in gene regulatory networks involved in brain development and adult neuroplasticity^9^. MiR-124a and miR-9 regulate the differentiation of embryonic stem (ES) cells into neurons or glial cells^10^. while brain-specific miR-9 plays a crucial role in regulating the cellular behavior of stem-cell-derived neural precursor cells (NPCs)^11^. Studies have shown that miRNAs, through epigenetic regulation, are vital participants in nervous system diseases such as convulsions and epilepsy.

In previous studies, miR-148a-3p has been found to be closely associated with non-small-cell lung cancer, oral cancer, and other cancers. For example, in non-small-cell lung cancer, miR-148a-3p forms a regulatory pathway with DNA methyltransferase 1 (DNMT1) to increase tumor proliferation^12^. In acute pancreatitis, miR-148a-3p can inhibit cell necrosis by targeting PTEN^13^. In addition, miR-148a-3p can affect apoptosis through targeted regulation of KLF6^14,15^. Furthermore, studies have shown that miR-148a-3p is processed from the precursor of miR-148a, and therefore has activity similar to that of miR-148a^16^. MiR-148a reduces liver damage in mice by regulating calmodulin-dependent protein kinase II^17^. New evidence has shown that miR-148-a may regulate the MAPK pathway during oxygen and glucose deprivation-induced microglial inflammation, which is of vital importance in ischemic injury induced by hypoxia. Recent studies have suggested that miR-148a-3p may play an important role in nervous and immune systems^18–21^. Compared with the diverse mechanisms for miR-148a-3p functions in cancer or other diseases, how miR-148a-3p regulates specific neuroimmune diseases is poorly understood. Previously, the apoptosis of hippocampal neurons was affected in brain injury by regulating the PI3K/Akt signaling pathway^22^. However, the exact role of miR-148a-3p and its underlying mechanism with the neuronal apoptosis and the effect of the PI3K/Akt signaling pathway in a recurrent FS model has not been clearly explored.

In this report, we investigated the pathogenic roles of miR-148a-3p in developmental convulsive brain injury. We used miRNA microarray to detect differences in miRNA expression in the hippocampal tissue between FS model and normal rats. We used a series of methods for data mining, information screening and analysis of miRNAs associated with the nervous system, and we discuss the roles of miR-148a-3p and its target gene Synaptojanin-1 (SYNJ1) in seizure-related brain damage during development. Our findings will help to elucidate the pathogenic factors of FSs and the subsequent development of epilepsy and provide new insights for the clinical diagnosis and treatment of seizure diseases.

## Results

### Differentially expressed miRNA in a rat model of recurrent FS

**Figure 1:**
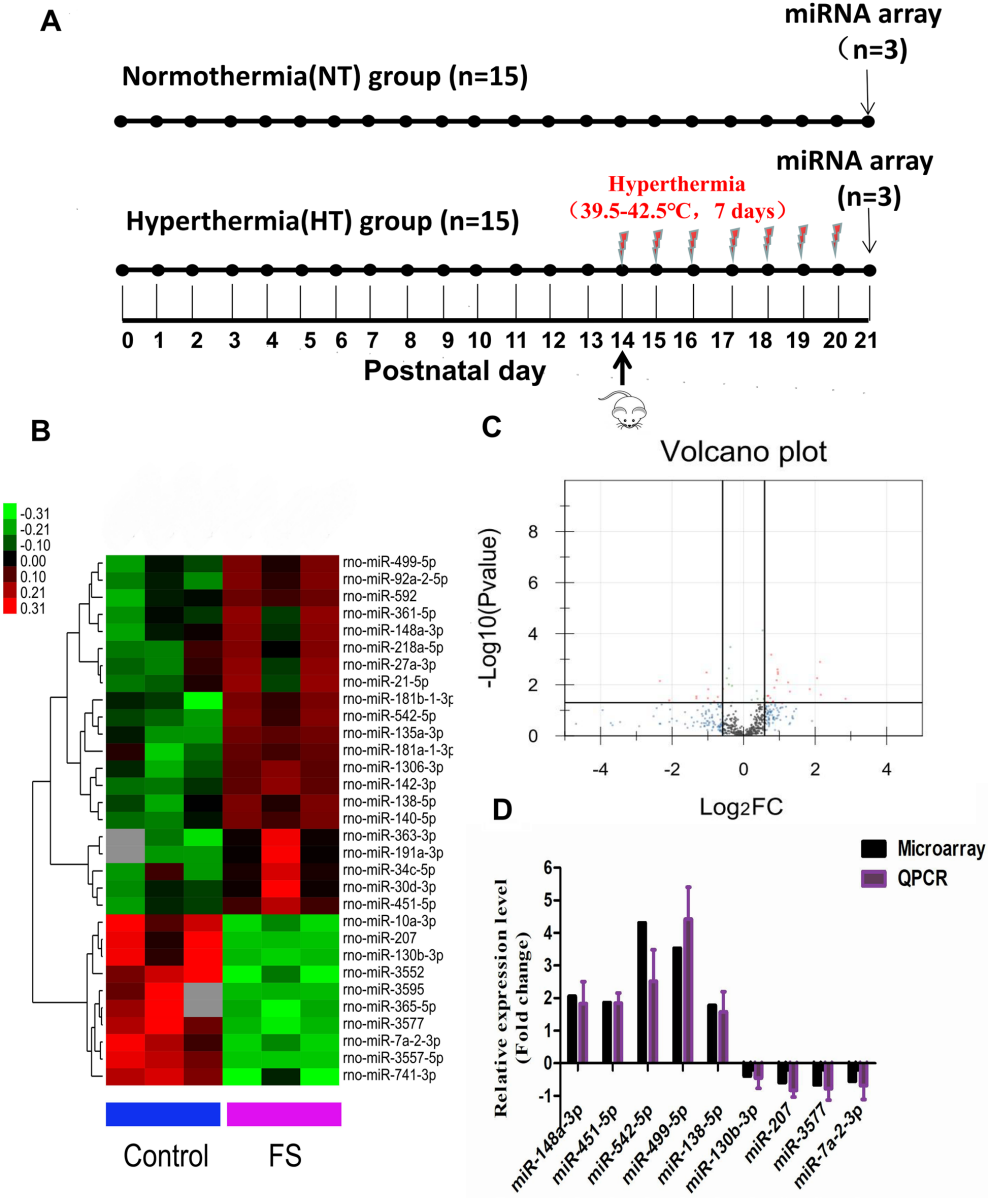
miRNA expression profile in a rat model of recurrent FS. (**A**) Experimental paradigms. Rats were treated with a hot-water bath at P14 for 7 consecutive days in the Hyperthermia (HT) group (bottom). (NT) group (upper) and (HT) group rats were killed at P21, and hippocampal tissues were taken for miRNA microarray. (**B**) Hierarchical clustering analysis. Heat map of the differentially expressed miRNAs between the HT and NT groups. A total of 31 miRNAs (P < 0.05, FC > 1.5) were identified from the hierarchical cluster analysis, including 21 upregulated miRNAs and 10 downregulated miRNAs. miRNA expression profiles are shown in the heatmap as upregulated (red color), downregulated (green color) and no change (black color). (**C**) Volcano plot analysis. (**D**) QRT-PCR verification. Nine differentially expressed miRNAs were verified in the HT group and the NT group.

To investigate how FSs affect miRNA expression profiles in the developing brain, we placed rats at postnatal day 14 (P14 rats) under hyperthermic conditions at 39.5–42.5 ℃ (HT group) (Fig. 1A). In addition, the normothermic group (NT) was not exposed to hyperthermic conditions.

Hierarchical clustering was performed based mainly on the similarity of the gene expression profile data; Pearson correlation coefficients were used as the measures of similarity. Finally, the individual samples were analyzed by cluster analysis. After hierarchical cluster analysis, all the experimental samples were divided into two main clusters that were consistent with our expected results based on the experimental grouping. Based on a P-value of < 0.05 and a fold change (FC > 1.5), 31 differentially expressed miRNAs were identified, including 21 upregulated miRNAs and 10 downregulated miRNAs (Fig. 1B,C). All the information is shown in Table S1.

To further validate the altered expression of miRNAs as detected by miRNA microarray, we selected nine differentially expressed miRNAs and verified them by qRT-PCR. The results of quantitative PCR were consistent with those of the microarray, confirming the value of the microarray as a fast, high-throughput, and large-scale gene expression research technique (Fig. 1D). The RT-PCR primer sequences are shown in Table S2.

### GO and pathway analysis of the miR-148a-3p target genes

**Figure 2:**
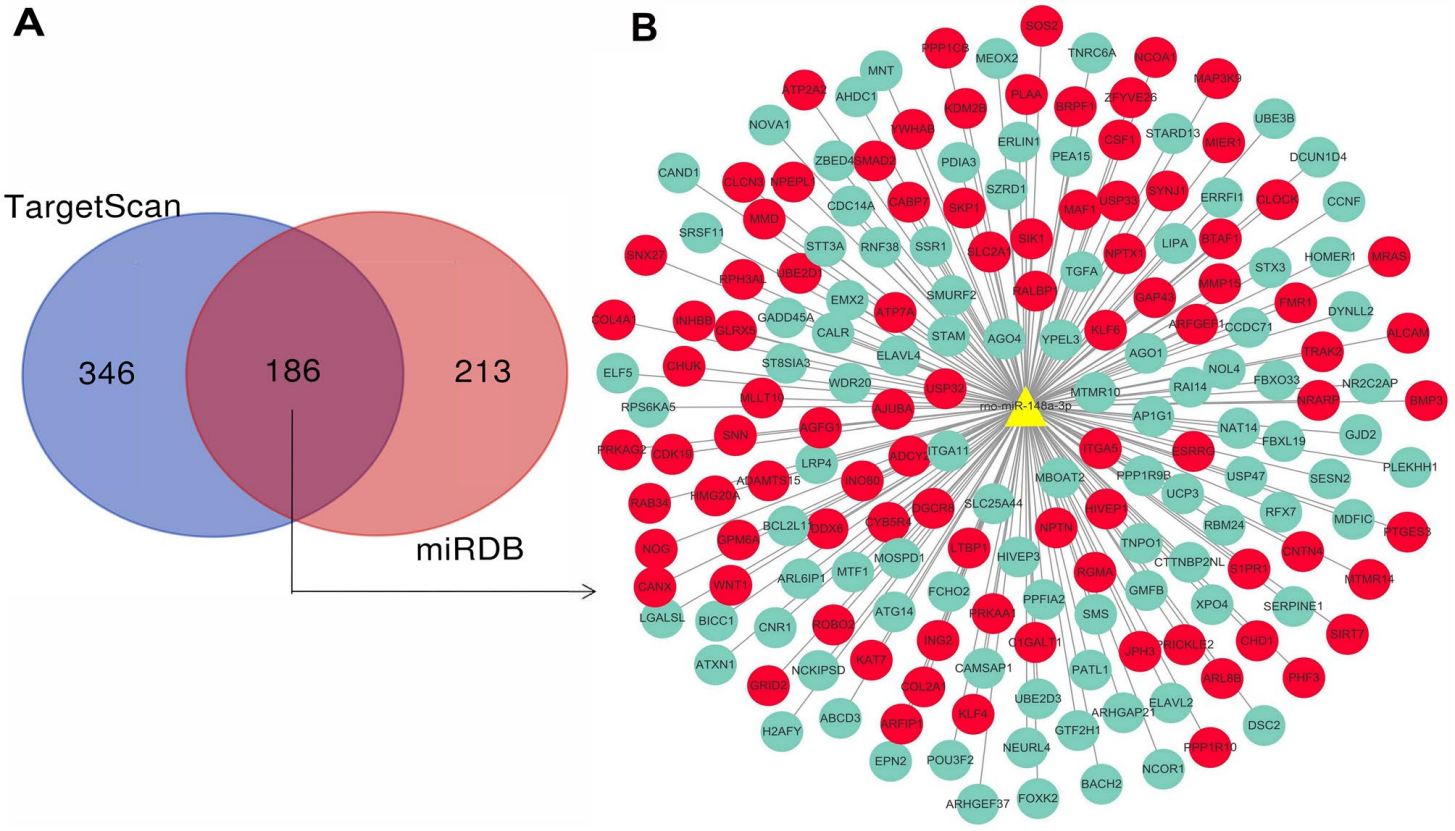
MiR-148a-3p target gene analysis. (**A**) Venn diagram of target genes for candidate miRNAs. (**B**) miR-148a-3p target gene network. Each node represents a target gene. Among them, red nodes represent genes related to nerve, immune and epigenetic pathways. Green nodes represent genes with other functions (development, gene transcription, etc).

Since miR-148a-3p has vital roles in the regulation of both the nervous and immune systems, we predicted the target genes of the miRNAs using the TargetScan and miRDB databases. A Venn diagram showed that 186 genes were found to be targeted by miR-148a-3p (Fig. 2A). The miR-148a-3p target genes are listed in Table S3. In addition, we selected candidate target genes related to nerve, immune, and epigenetic functions, which may act as key players in FS (Fig. 2B).

**Figure 3:**
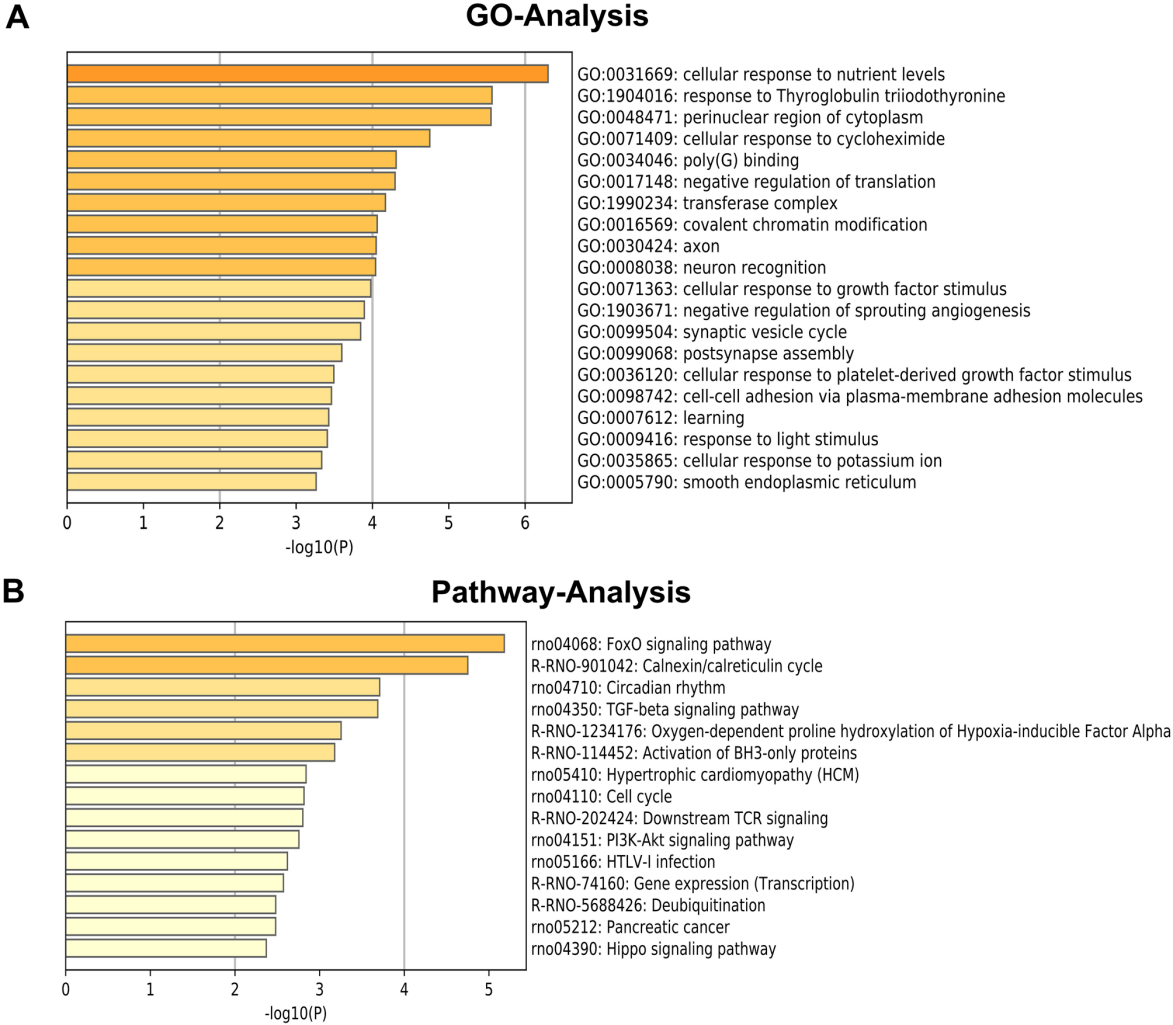
Gene ontology (GO) and pathway analysis for miR-148a-3p target genes. (**A**) Indicate biological process (BP), cellular component (CC) and molecular function (MF) predicted miR-148a-3p target genes. The vertical axis shows the GO terms, and the horizontal axis shows the − log10(P). (**B**) Significant pathways for predicted miR-148a-3p target genes. The vertical axis shows the pathway categories, and the horizontal axis shows the enrichment scores of pathways. P < 0.05 was used as a threshold to select significant GO categories and KEGG pathways. − Log 10p was the base 10 logarithm of the P-value.

The GO functions of the genes were classified according to the NCBI and AmiGO databases. The significant GO functional classifications obtained mainly included cellular response to nutrient levels, cellular response to starvation, perinuclear region of cytoplasm, covalent chromatin modification, axon, neuron recognition, and synaptic vesicle cycle (Fig. 3A). Furthermore, the significant pathways of the target genes were analyzed according to the screening criterion of P < 0.05. Through the KEGG, BioCarta, Reactome and other databases, the top 10 signaling pathways with significant differences were obtained by Fisher's exact test of hypergeometric distributions. Significant pathways mainly included the FoxO signaling pathway, Calnexin/calreticulin cycle, TGF-β signaling pathway, PI3K-AKT signaling pathway, downstream TCR signaling, and the hippocampal signaling pathway (Fig. 3B).

### PPI network construction and module and hub gene identification

**Figure 4:**
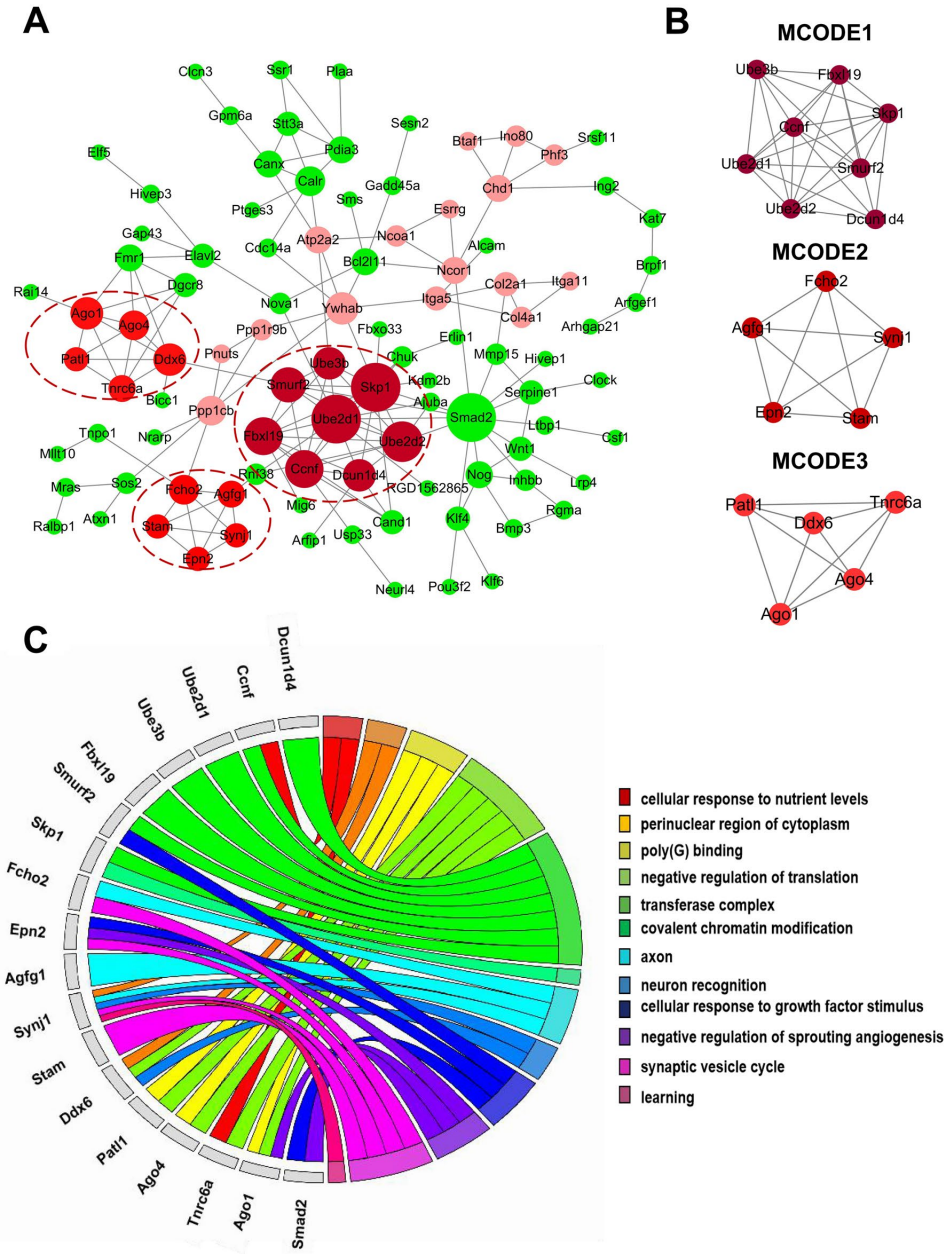
PPI network analysis and functional enrichment analysis of hub genes. (**A**) PPI network analysis. Circular nodes represent the miR-148a-3p target genes. Circular nodes in red represent the hub genes. A larger node size corresponds to more genes that are co-expressed with the gene. Darker colors indicate greater significance. (**B**) Three significant modules selected from the PPI network. Each node represents a protein. (**C**) GO enrichment analysis of 18 hub genes. Different color bands represent different GO terms.

Protein interaction networks have proven to be powerful tools for predicting and mining new essential genes in specific GO and KEGG pathways. Using the STRING online database and Cytoscape software, a total of 186 miR-148a-3p target genes were filtered into the PPI network complex (Fig. 4A).

Subsequently, the PPI network of miR-148a-3p target genes was constructed with the most important module obtained based on the MCODE analysis in Cytoscape (Fig. 4B). When “Node Score ≥ 0.2” was defined as the cut-off criterion in MCODE, three clusters were identified from the PPI network, and the most significant cluster consisted of 8 nodes and 26 edges. The 18 node degree genes were DCUN1D4, CCNF, UBE2D2, UBE2D1, UBE3B, FBXL19, SMURF2, SKP1, FCHO2, EPN2, AGFG1, SYNJ1, STAM, DDX6, PATL1, AGO4, TNRC6A, and AGO1. Furthermore, we analyzed the functions of the 18 genes, 6 of which (FCHO2, SYNJ, AGFG1, DDX6, STAM, and EPN2) are mainly involved in axon, neuron recognition, and synaptic vesicle cycle (Fig. 4C). These findings may play a particularly important role in FS.

### Effects of different concentrations of KA on the apoptosis rate of hippocampal neuronal cells in vitro

Many researches have shown that kainic acid (KA) was uesed to induce hippocampal neuronal apoptosis, and it has been widely used as an experimental drug for seizures models since then^23^. Specifically, KA has been shown to activate glutamate receptors, affect mitochondria function, and cause cell death in neuronal cells^24^.

Rat hippocampal neurons in culture were treated with 50, 100, 150 and 200 μM KA for 24 h. The cells were collected, and the KA-induced apoptosis of the hippocampal neurons was observed by TUNEL staining. The results showed that the number of apoptotic hippocampal neurons and the apoptosis rate of hippocampal neurons in the 50 μM KA group were significantly higher than those in the control group; the numbers of apoptotic cells and the apoptosis rates were further increased in the 100, 150 and 200 μM groups, and the apoptosis rates changed in a dose-dependent manner (Fig. S1). However, higher doses of KA induce greater neurotoxicity, which can lead to the death of large numbers of nerve cells. Therefore, in the follow-up experiment, we selected 100 μM KA for treatment of neuronal cells to establish an in vitro seizure model.

### Sub-cellular location of miR-148a-3p in vivo and vitro

**Figure 5:**
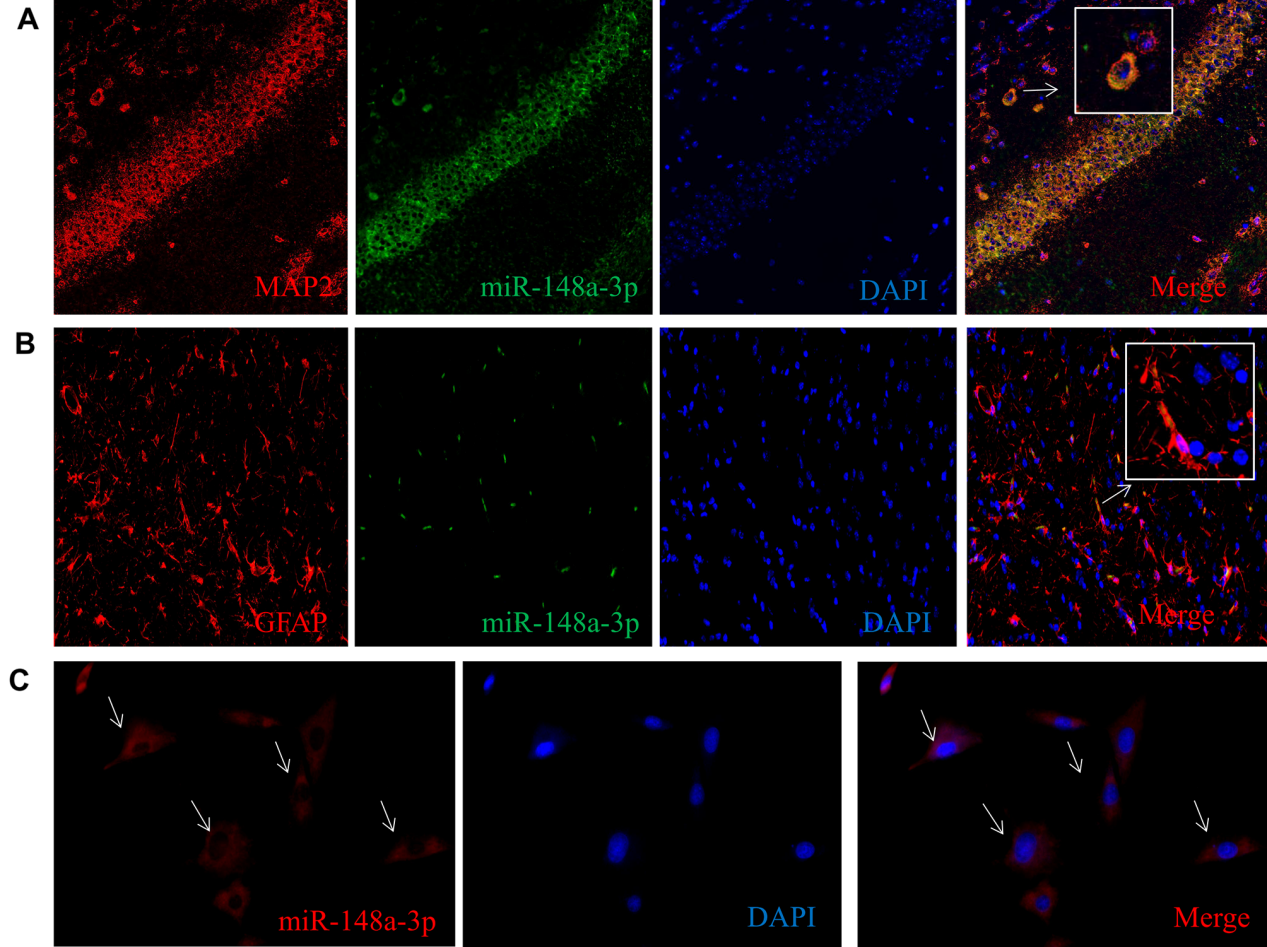
Sub-cellular location of miR-148a-3p in vivo and vitro. (**A**–**C**) Cellular location of miR-148a-3p in neuronal cells in normal rat hippocampus tissue. (**D**–**F**) Cellular location of miR-148a-3p in astrocyte cells in normal rat hippocampus tissue. (**G**–**I**) MiR-148a-3p was localized in the cytoplasm of H19-7 hippocampal neuronal cells.

To further study the function of miR-148a-3p, we measured the cellular localization of miR-148a-3p in vivo and vitro and found that miR-148a-3p was colocalized with MAP2 (neuronal maker) and GFAP (astrocytes maker) (Fig. 5).

### SYNJ1 is a target gene of miR-148a-3p

**Figure 6:**
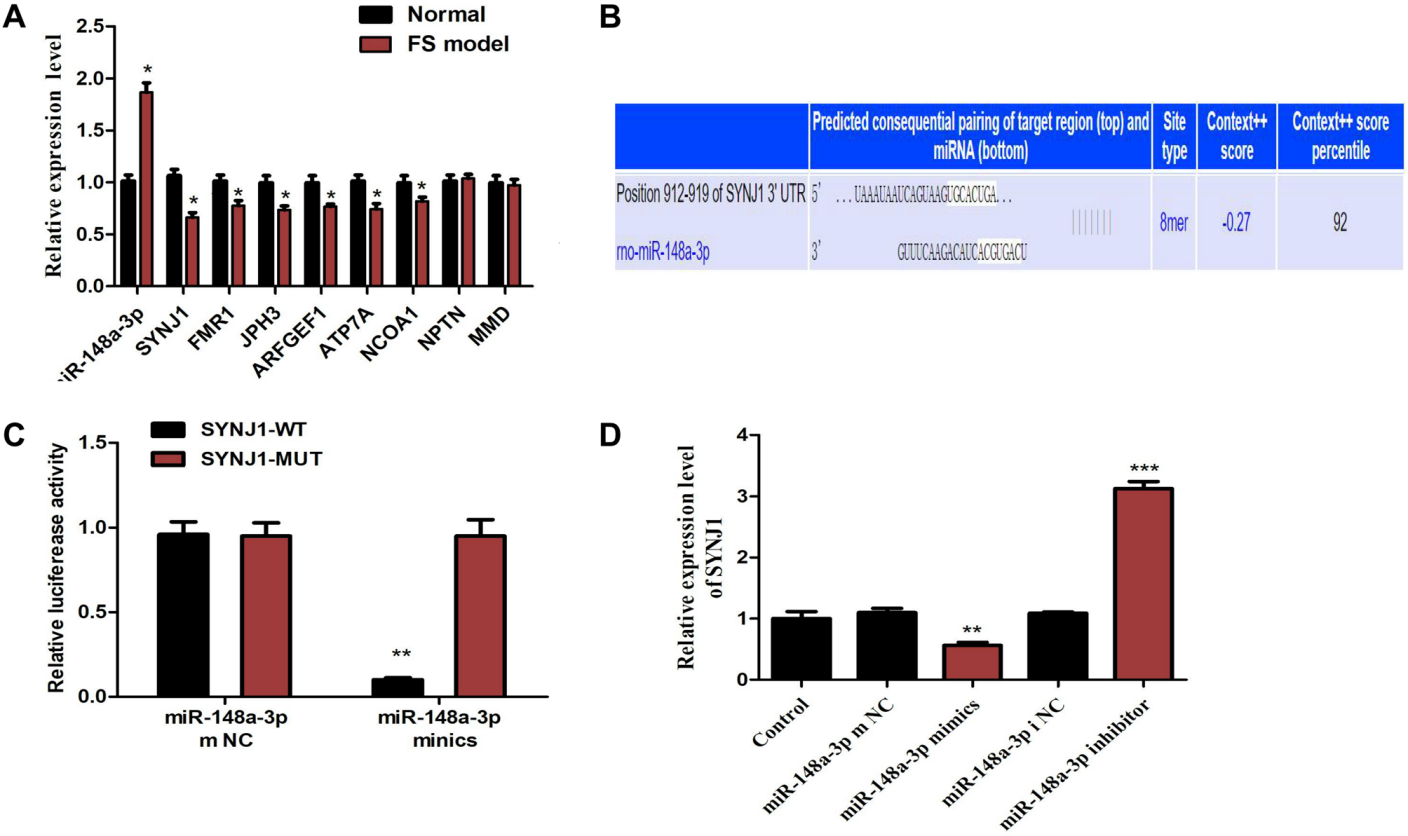
SYNJ1 is a bona fide target of miR-148a-3p in rat hippocampal neuronal cells. (**A**) Detection of predicted miR-148a-3p target genes between the normal and FS model groups. Relative mRNA expression of the miR-148a-3p target genes. *P < 0.05 compared with the normal group. (**B**) Predication that SYNJ1 is a target gene of miR-148a-3p by TargetScan. (**C**) Dual-luciferase experiment to verify the targeting relationship between miR-148a-3p and SYNJ1; **P < 0.01 vs. NC group vs. miR-148a mimics-3p + WT-SYNJ1 group; ^##^P < 0.01 vs, miR-148a-3p mimics + WT-SYNJ1 group vs. miR-148a-3p mimics + MUT-SYNJ1 group. The data were analyzed by one-way ANOVA; the pairwise comparison after ANOVA was analyzed by LSD-t test. (**D**) Relative SYNJ1 levels after different treatments. **P < 0.01, ***P < 0.001 vs. control group.

To screen the target genes of miR-148a-3p, which is closely related to the neuroimmune system, we detected the predicted target genes by qRT-PCR. We found that the FS model group significantly increased the expression of miR-148a-3p and significantly decreased the mRNA expression of miR-148a-3p target genes, mainly including SYNJ1, FMR1, JPH3, ARFGEF1, ATP7A, NCOA1(Fig. 6A). These genes are involved in the nervous and immune systems. Meanwhile, significantly decreased Expression of SYNJ1 was detected in the hippocampal neurons after KA-induced. In addition, the SYNJ1 gene plays a vital role in the function and pathways of the nervous and immune systems^25^, including Parkinson’s^26^, seizures^27^, epilepsy. Based on the reasons above, SYNJ1 was selected as a candidate target gene for verification.

The target relationship between miR-148a-3p and SYNJ1 is displayed in Fig. 6B, which shows that miR-148a-3p was able to target SYNJ1. To confirm that SYNJ1 was a direct target gene of miR-148a-3p, we performed a double fluorescein enzyme reporter experiment. Recombinant plasmids of wild-type (WT)-miR-148a-3p/SYNJ1 and mutated (Mut)-miR-miR-148a-3p/SYNJ1 were constructed by inserting the luciferase reporter vector and the wild-type or mutant 3′ UTR sequence of SYNJ1. WT-miR-148a-3p/SYNJ1 and Mut-miR-148a-3p/SYNJ1 vectors were co-transfected with miRNA-148a-3p mimic or control plasmids into hippocampal neurons. The results showed that compared with that of the control plasmid group, the luciferase activities of the miR-148a-3p mimic and WT-pGL3-SYNJ13′-UTR cotransfection group were inhibited, while the luciferase activities of the miR-148a-3p mimic and MUT-pGL3-SYNJ13′-UTR co-transfection group were not significantly different (Fig. 6C). In addition, as we expected, the SYNJ1 mRNA level was changed in response to treatment with miR-148a-3p mimics or inhibitors. MiR-148a-3p mimic treatment reduced the mRNA level of SYNJ1. Conversely, miR-148a-3p inhibitors caused accumulation of SYNJ1 (Fig. 6D). Taken together, these results show that SYNJ1 is a novel bona fide target of miR-148a-3p in rat hippocampus neuronal cells.

### MiR-148a-3p promotes cell apoptosis of hippocampal neurons after KA-induction

To further confirm the correlation between miR-148a-3p level and KA-induced neuron cell apoptosis, we manipulated miR-148a-3p levels by treating H19-7 rat hippocampal neuronal cells with either miR-148a-3p mimics or miR-148a-3p inhibitors, followed by 100 μM KA treatment in vitro.

**Figure 7:**
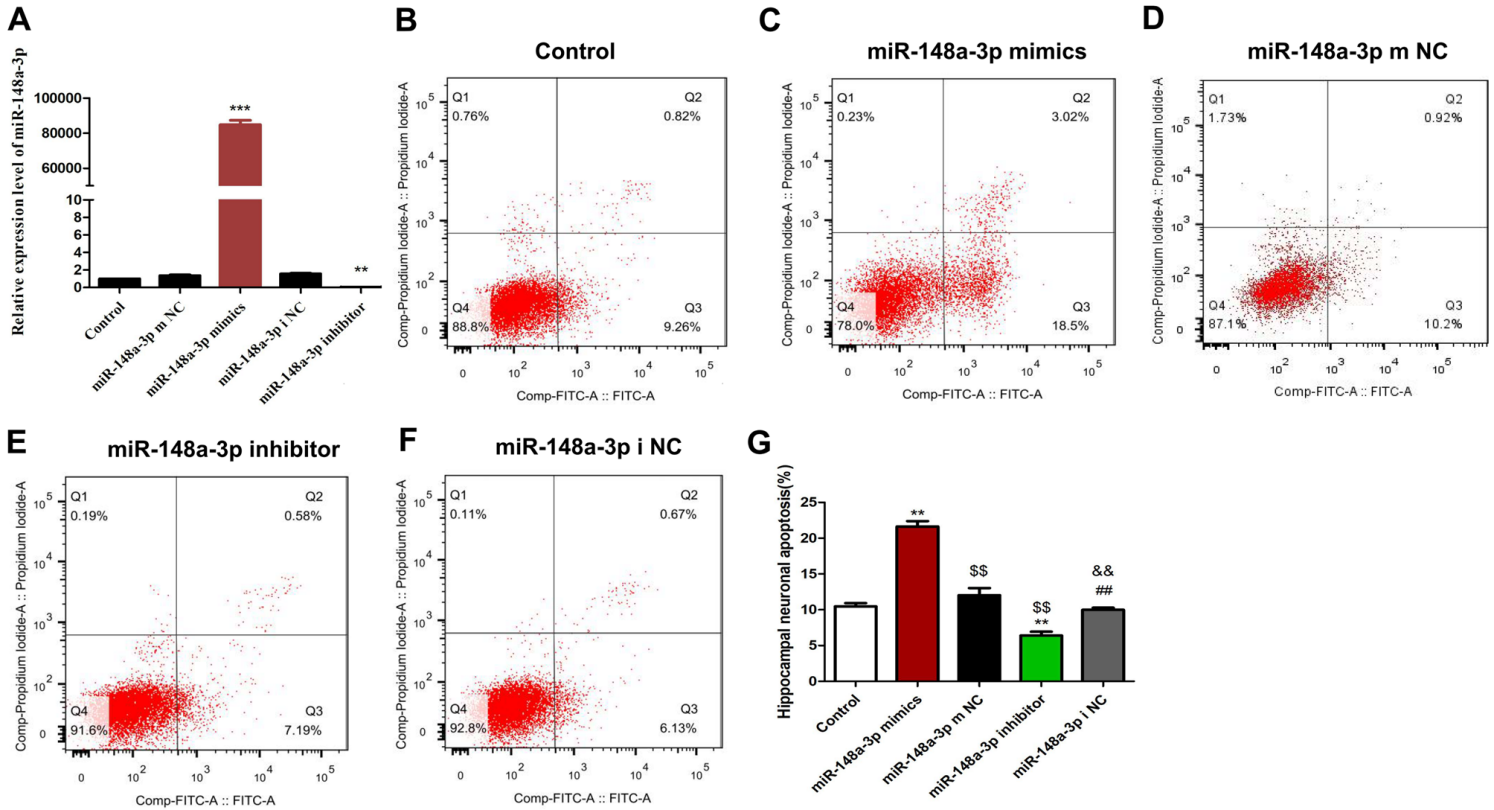
Inhibition of miR-148a-3p reduces hippocampal neuronal apoptosis by kainic acid. (**A**) Relative miR-148a-3p levels after different treatments. (**B**–**F**) Cell apoptosis detection by flow cytometry. (**G**) Apoptosis rates of hippocampal neurons. Data are shown as the Mean ± S.E.M. and represent three independent experiments. **P < 0.01 vs. control group; ^$$,##^P < 0.01 vs. miR-148a-3p mimics group; ^&&^P < 0.01 vs. miR-148a-3p inhibitor group.

As shown in Fig. 7A, we first determine the effectiveness of miR-148a-3p mimics or inhibitor for the expression of miR-148a-3p. The results showed that the miR-148a-3p mimics efficiently increased miR-148a-3p levels (P < 0.001) and the miR-148a-3p inhibitor exhibited the lowest miR-148a-3p expression (P < 0.01).

As shown in Fig. 7B–G, we found that the apoptosis rate of hippocampal neuronal cells transfected with the miR-148a-3p mimic was significantly higher than that of hippocampal neurons transfected with the control construct (P < 0.01), while that of hippocampal neurons transfected with the miR-148a-3p inhibitor was significantly lower (P < 0.01). Compared with that of the miR-148a-3p mimic group, the apoptosis rates of the miR-148a-3p mimic NC group and the miR-148a-3p inhibitor group were significantly decreased (P < 0.01). In addition, compared with that of the miR-148a-3p inhibitor NC group, the apoptosis rate of the miR-148a-3p inhibitor group was significantly decreased (P < 0.01). All findings strongly support our hypothesis that miR-148a-3p mediates neuronal cell apoptosis in response to KA treatment in vitro.

### MiR-148a-3p mediates hippocampal neuronal apoptosis by targeting SYNJ1 after KA-induction

**Figure 8:**
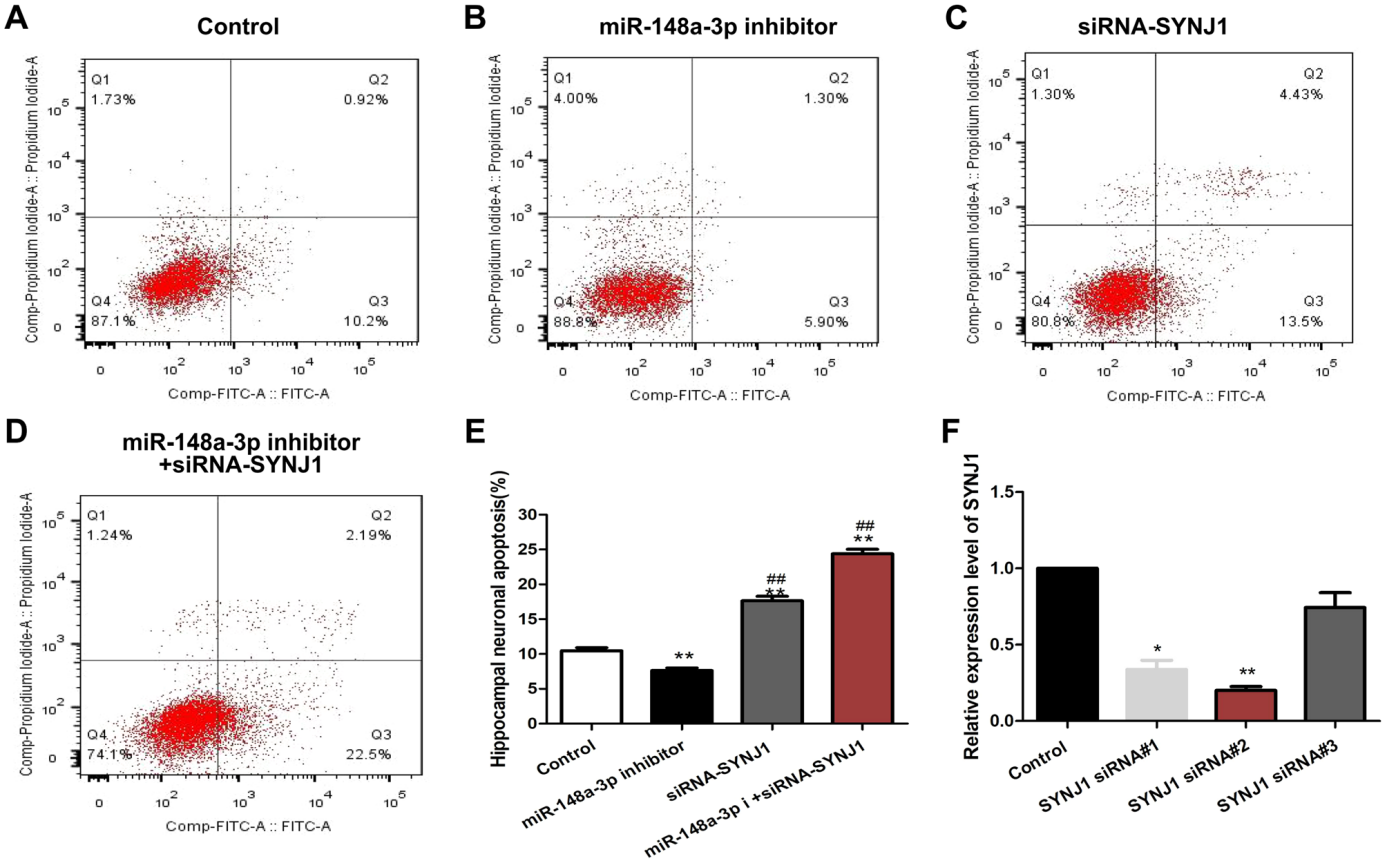
miR-148a-3p regulation of hippocampal neuron apoptosis through targeting SYNJ1. (**A**–**D**) Hippocampal neuron apoptosis using flow cytometry. H19-7 hippocampal neuronal cells were incubated with 100 µM KA for 48 h. miR-148a-3p inhibitor and siRNA-SYNJ1 were added to the culture 4 h prior to KA treatment. Cells were collected and stained with Annexin V and propidium iodide (PI) and then examined by flow cytometry. (**E**) Hippocampal neuron apoptosis rate. Results are shown as the mean ± S.E.M. and represent three independent experiments. **P < 0.01 vs. control group; ^##^P < 0.01, vs. miR-148a-3p inhibitors group. (F) Comparison of the effects of three different SYNJ1 siRNAs. *P < 0.05, SYNJ1 siRNA#1 vs. control group; **P < 0.01, SYNJ1 siRNA#2 vs. control group.

To further confirm whether miR-148a-3p depends on the SYNJ1 to regulate the apoptosis of hippocampal neurons, we studied the effect of SYNJ1 siRNA on the miR-148a-3p-mediated apoptosis of hippocampal neurons treated with KA. We first detected the silencing efficacy of SYNJ1-siRNA. As shown in the RT-qPCR results in Fig. 8F, compared with the control, the siRNA-1, siRNA-2, and siRNA-3 groups had decreased mRNA expression levels of SYNJ1 while the siRNA-2 group exhibited the lowest SYNJ1 expression (P < 0.01). These findings demonstrated that the silencing efficacy was highest in the siRNA-2 group; thus, sequences in the siRNA-2 group were selected for cell transfection.

After successful transfection of rat hippocampal neurons with the miR-148a-3p inhibitor and miR-148a-3p inhibitor + SYNJ1 siRNA for 48 h, the cells were treated with 100 μM KA and cultured for 24 h. The cells were collected, and hippocampal neuronal apoptosis was detected by flow cytometry. The results showed that the apoptosis rate of hippocampal neurons after miR-148a-3p inhibitor transfection was significantly decreased compared to that of the control (P < 0.01). Compared with that of the miR-148a-3p inhibitor group, the apoptosis rate of the miR-148a-3p inhibitor + SYNJ1 siRNA group was significantly increased (P < 0.01) (Fig. 8A–E). Taken together, our results indicate that SYNJ1 acts as a downstream target to mediate miR-148a-3p regulation of apoptosis in rat hippocampus neuronal cells.

### MiR-148a-3p mediates hippocampal neuronal apoptosis via activation of PI3K-AKT signaling pathway

**Figure 9:**
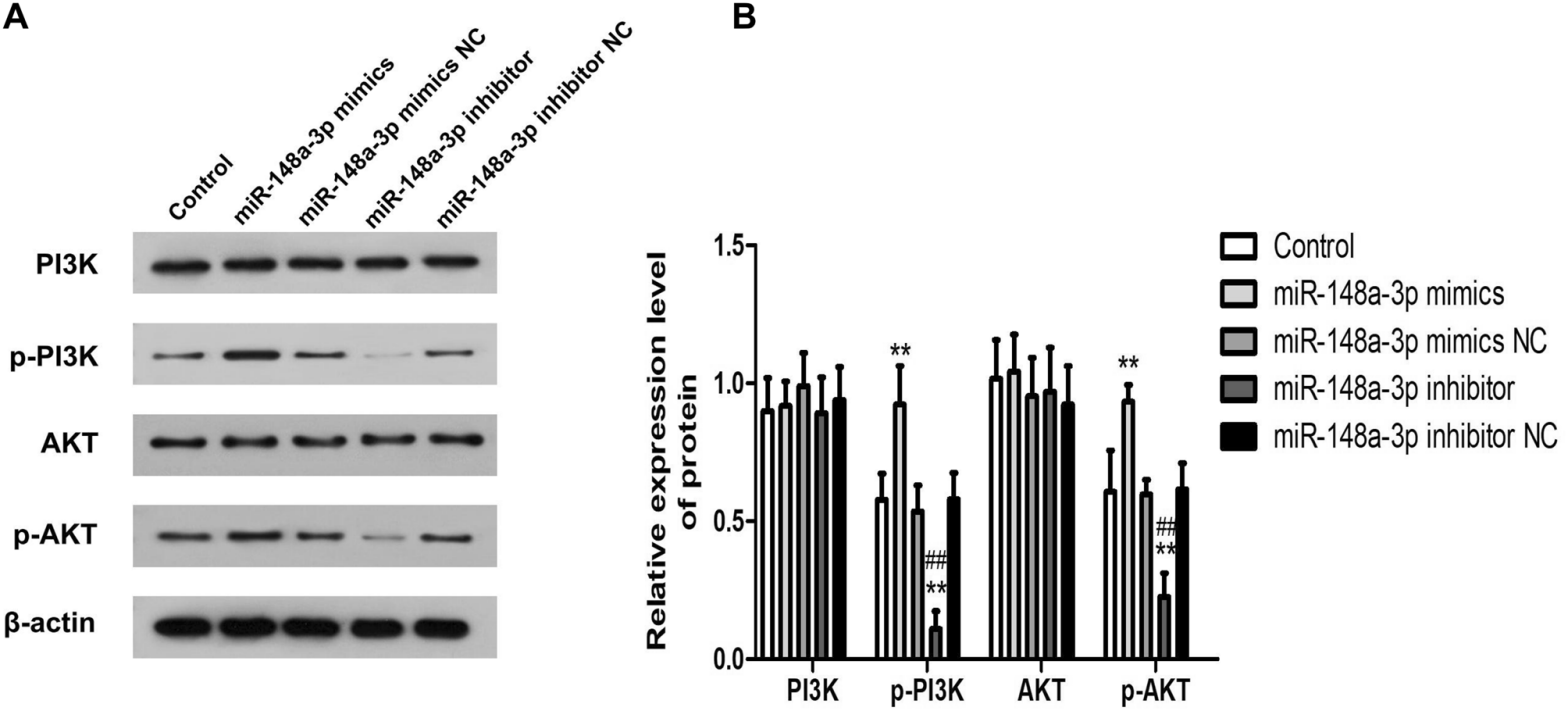
Protein expression level of PI3K/Akt signaling pathway-related proteins in hippocampal neurons of rats. (**A**) protein bands of PI3K/Akt signaling pathway-related proteins detected by western blotting (**B**) Comparison on expression of PI3K/Akt signaling pathway-related proteins. **P < 0.01 compared with the control group; ^##^P < 0.01 compared with the miR-148a-3p mimics group. PI3K hosphatidylinositol 3-kinase, p-PI3K phosphorylated PI3K, p-Akt phosphorylated Akt.

To further study whether miR-148a-3p can activate the PI3K/AKT pathway to regulate the apoptosis of hippocampal neurons, western blotting was performed to determine protein expression level of related proteins of the PI3K/Akt signaling pathway (PI3K, p-PI3K, Akt, and p-Akt) (Fig. 9). and the results indicated that miR-148a-3p could promote the activation of the PI3K/Akt signaling pathway in hippocampal neurons.

The protein expression of PI3K and Akt was not significantly different in hippocampal neurons of all the groups (all P > 0.05). In comparison to the control group, the protein expression of p-PI3K and p-Akt were significantly elevated in miR-148a-3p mimics group (P < 0.01), while that in miR-148a-3p inhibitor group was significantly decreased (P < 0.01). The protein expression of p-PI3K and p-Akt was not significantly different in hippocampal neurons of mimics NC and inhibitor NC groups (all P > 0.05).

Compared with the miR-148a-3p mimics group , the protein expression of p-PI3K and p-Akt were significantly decreased in the miR-148a-3p inhibitor group (P < 0.01), which indicated that miR-148a-3p expression promoted the activation of the PI3K/Akt signaling pathway in hippocampal neurons after KA-induced.

## Discussion

FSs are most common in children at the age of 5, and a variety of factors may increase the risk of FSs^28^. MiRNAs may play an important role in neural circuitry by functioning in various brain regions and may also play key roles in neurological diseases, such as seizures and epilepsy^29–31^. Among these miRNAs, miR-148a-3p is involved in the development and dynamic balance of the neuron-immune system and in various types of cancers, autoimmune diseases, inflammatory diseases, and other pathological processes^32,33^. Therefore, the primary purpose of this study was to explore the potential mechanism by which miR-148a-3p contributes to FSs to better elucidate the pathological mechanism of FSs. This study provided evidence that downregulation of miR-148a-3p inhibited apoptosis of hippocampal neurons after KA induced through the PI3K/Akt signaling pathway.

In this report, we first established a rat model of recurrent FSs and used miRNA expression profile chip technology to analyze the hippocampi of rats in the recurrent convulsion group and the normal group. According to the results of hierarchical cluster analysis, the six groups of experimental samples were divided into two different clusters, from which it was inferred that the experimental sample classification was reliable and consistent with the expected results. Through information analysis, 31 differentially expressed miRNAs and related functional information were obtained. Twenty-one miRNAs were significantly upregulated, including miR-542-5p, miR-499-5p, miR-148a-3p, and miR-451-5p. Eleven miRNA genes were significantly downregulated, including miR-207 and miR-130b-3p. Whether these differentially expressed miRNAs participate in the development of FSs needs further study. In addition, to verify the reliability of the gene chip data, the expression profiles of 9 differentially expressed miRNAs were verified by qRT-PCR, and the results were consistent with the chip results, providing a solid foundation for the subsequent expression profile analysis.

Information mining and comprehensive analysis revealed that the expression level of miR-148a-3p was significantly increased in the hippocampus in the FS models, consistent with the results of qRT-PCR. Therefore, we selected miR-148a-3p for in-depth research and analysis. Studies have shown that the function of differentially expressed miR-148a-3p is closely related to neuroimmunity and apoptosis. Recent studies have shown that in ischemic stroke-related diseases, miR-148a-3p can interact with lncRNA-h19 and ROCK2 genes to form a pathway that regulates the oxidative stress caused by ischemic stroke^18^. Additionally, miR-148a-3p can target the KLF6 gene to regulate the proliferation and apoptosis of skeletal muscle cells^14,15^. As the precursor of miR-148a-3p, miR-148a has the same activity^34^. Clinical studies have shown that there are significant abnormalities in the expression of miR-148a in patients with neurological diseases such as Parkinson's disease and Alzheimer's disease. Other differentially expressed miRNAs screened by bioinformatics analysis are also worth studying. Previous studies have revealed that the functions of these miRNAs are closely related to neuroimmune and other factors. For example, studies have shown that miR-499-5p can play a neuroprotective role by regulating the expression of C-reactive protein in hypoxic-ischemic encephalopathy^35^. MiR-207 may be involved in cognitive impairment induced by obstructive sleep apnea (OSA)^36^. Furthermore, miR-130b-3p can inhibit the inflammatory response induced by eCIRP^37^.

We predicted the target genes of miR148a-3p with TargetScan and miRDB, and a total of 186 overlapping target genes were obtained. A target gene relationship map was constructed using the relevant database information. The diagram confirmed that the DNMT1 gene is a target of miR148a-3p and that there is a close relationship between DNMT1 and the occurrence and development of epilepsy^38^. In addition, epigenetic regulation plays vital roles in nervous system diseases^39^. Among the predicted target genes were many genes strictly related to neuroimmune function, such as SYNJ1, Noggin (NOG), Neuronplastin (NPTN), Nuclear receptor coactivator 1 (NCOA1), and SOS Ras/Rho guanine nucleotide exchange factor 2 (SOS2). Previous studies have shown that the NOG gene can restore stem cell populations and neurogenesis in the hippocampus^40^. NOG gene can also promote the repair of myelin sheaths^41^. NPTN, a multifunctional neuronal adhesion molecule, is closely related to long-term synaptic plasticity^42^.

Subsequently, we selected the potential target genes of the significantly upregulated miR-148a-3p and analyzed their GO functions and KEGG signaling pathways. The different GO term and KEGG pathway information suggests that there is a close relationship between FSs and neuroimmunity, which provides an essential clue for further study of the role of immune inflammation in FS. The results of GO analysis showed that the functions of the potential target genes were closely related to nervous system processes, such as neuroprotection, neuron migration, and hippocampal signaling pathways. Recent studies have shown that immune and inflammatory processes play essential roles in FSs, especially innate immune system processes and subsequent inflammatory responses caused by infection, nerve trauma, and other factors related to epilepsy. Consistent with these roles, the insulin signaling pathway was significantly enriched. Studies have shown that the insulin signaling pathway is closely related to epilepsy. For example, administration of low-dose intranasal insulin can reduce the frequency of spontaneous convulsions and epileptic discharges and increase the cognitive abilities of epileptic mice^43^. Additionally, under epileptic conditions, neuronal death induced by convulsions is affected by activation of the FoxO signaling pathway^44^. Furthermore, the FoxO signaling pathway family can participate in cell death in a variety of pathological conditions^45^. FoxO1 and FoxO2 play essential roles in the development and differentiation of immune cells. FoxO1 and FoxO3 can promote the formation of regulatory T cells and inhibit the formation of T-helper 1 (Th1) and T-helper 17 (Th17) cells^46^.

The PPI network displayed functional connections of miR-148a-3p target genes, and certain hub genes were selected based on the MCODE analysis. Most of them were enriched in neuron and synaptic function. Neurons and synaptogenesis factors have always played a vital role in FS. Based on the module analysis of the PPI network, 6 neural-associated genes were selected: SYNJ1, FCHO2, AGFG1, DDX6, STAM, and EPN2. Thus, our findings indicate that these screened key genes may be the driving forces underlying the occurrence of FS.

SYNJ1, a polyphosphate inositol phosphatase, exists in presynaptic nerve endings and protein complexes involved in cellular endocytosis and plays a vital role in the phosphorylation of synaptic vesicles. In addition, the SYNJ1 gene plays essential roles in the nervous and immune systems. SYNJ1 gene mutations are associated with two rare nervous system diseases: early-onset Parkinson's disease and severe neurodegeneration with refractory seizures and recurrent seizures^47^. Deletion of the SYNJ1 gene can lead to seizures^25^. SYNJ1 has been reported as a potential regulator of allogeneic T cell responses^48^. The level of SYNJ1 mRNA was reduced after allogeneic stimulation of naive T cells. Knockdown of SYNJ1 in allogeneically stimulated T cells confirmed its role in T cell proliferation and cytokine responses^48^. Through comprehensive analysis, we found that miR-148a-3p may affect the occurrence and development of convulsions through its potential target gene, SYNJ1. In a double luciferase experiment, it was shown that SYNJ1 can indeed be directly regulated by miR-148a-3p.

Previous studies have revealed that miRNAs are closely related to the apoptosis of hippocampal neurons. Upregulation of miR-223 expression can inhibit hippocampal neuronal apoptosis and brain damage in rats with convulsions^49^. MiR-421 can inhibit the apoptosis of hippocampal neurons in epileptic rats through the TLR signaling pathway^50^. Silencing miR-134 can reduce the damage to hippocampal neurons and the frequency of spontaneous convulsions in epileptic rats^51^. Other studies have shown that there is a close relationship between miR-148a-3p and apoptosis. MiR-148a-3p and DNMT1 form a regulatory pathway that increases tumor proliferation. Other studies have shown that miR-148a-3p can inhibit cell necrosis by bypassing PTEN targeting in acute pancreatitis^16^. In a previous study, we used miRNA arrays and qRT-PCR to confirm that hippocampal miR-148a-3p was significantly upregulated after recurrent convulsions during development, suggesting that miR-148a-3p may be involved in the occurrence and development of convulsions. To confirm that miR-148a-3p is involved in the apoptosis related to convulsion injury, we used rat hippocampal neurons (H19-7 cells) as the research objects in this study. We transfected hippocampal neurons with a miR-148a-3p mimic and a miR-148a-3p inhibitor and treated them with KA to establish a neurotoxicity model that approximated convulsions. We thus observed the effects of overexpression or low expression of miR-148a-3p on the apoptosis of hippocampal neurons treated with KA and investigated the related mechanisms. The results showed that compared with those in the non-transfected group, the miR-148a-3p expression levels in hippocampal neurons in the miR-148a-3p mimic group were significantly upregulated while those in the miR-148a-3p inhibitor group were significantly downregulated. Cell models of miR-148a-3p overexpression and low expression were thus successfully constructed and can be used for follow-up studies.

This report showed that the apoptosis rate of hippocampal neurons was significantly higher in the miR-148a-3p mimic group than in the control group while the apoptosis rate of hippocampal neurons was significantly lower in the miR-148a-3p inhibitor group than in the miR-148a-3p inhibitor group. It is suggested that overexpression of miR-148a-3p can promote KA-induced apoptosis of hippocampal neurons, while low expression of miR-148a-3p can inhibit apoptosis. To further confirm that miR-148a-3p regulates hippocampal neuronal apoptosis in a SYNJ1-dependent manner, we studied the effects of SYNJ1 siRNA on miR-148a-3p-mediated regulation of hippocampal neuronal apoptosis under KA treatment. Apoptosis was detected by flow cytometry. The apoptosis rate of hippocampal neurons in the SYNJ1 siRNA + miR-148a-3p inhibitor group was significantly higher than that in the SYNJ1 siRNA + miR-148a-3p mimic group, indicating that SYNJ1 siRNA transfection could block the decrease in hippocampal neuronal apoptosis induced by the miR-148a-3p inhibitor. These findings further confirmed that the effect of miR-148a-3p on hippocampal neuronal apoptosis depends on the existence of SYNJ1, which enables the regulation of hippocampal neuronal apoptosis after seizures.

**Figure 10:**
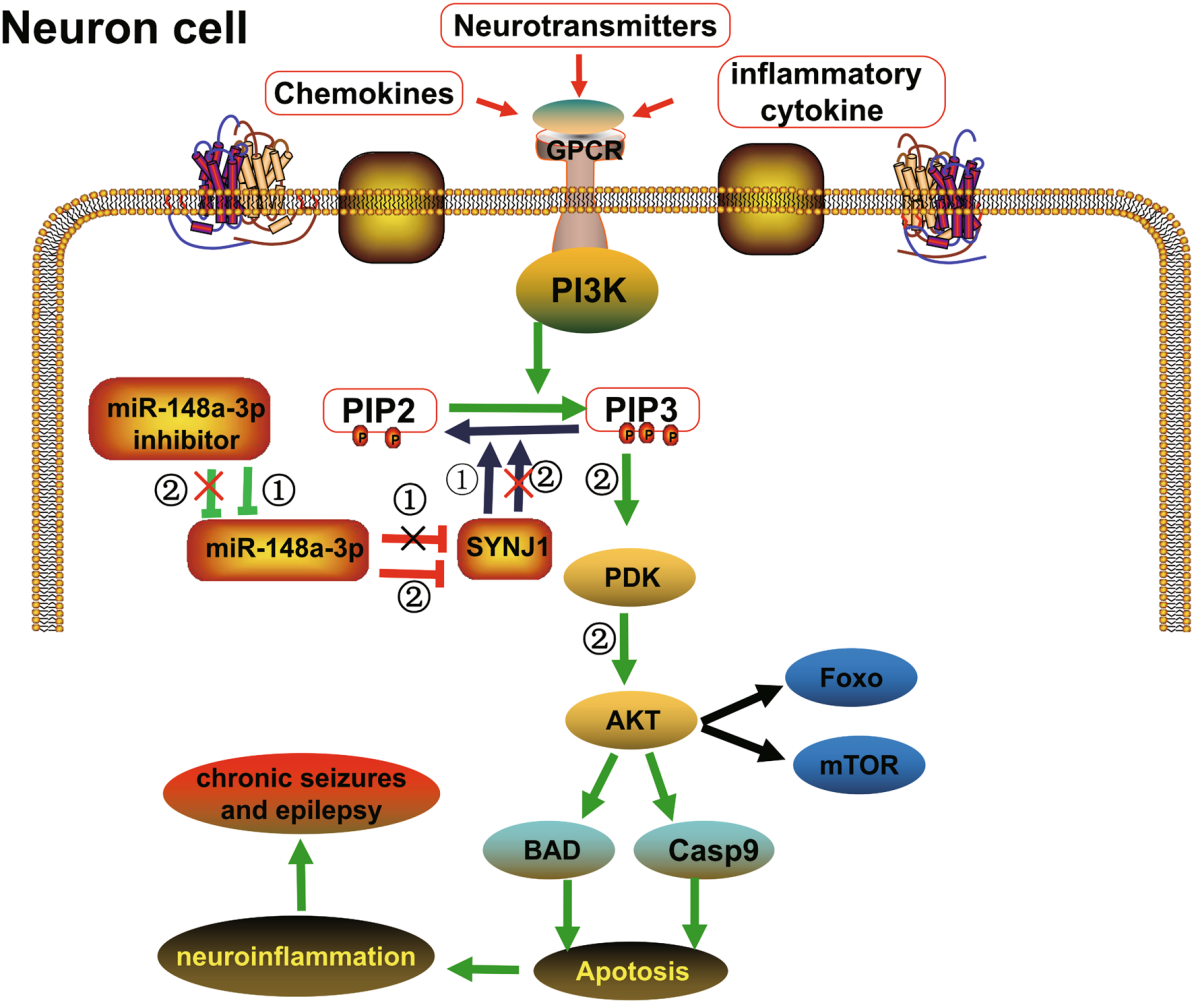
Schematic of the proposed hypothesis model showing that miR-148a-3p positively regulates neuronal apoptosis via activating the PI3K-AKT signaling pathway by targeting SYNJ1. In chronic seizure or epilepsy tissue, neurons and astrocytes and even microglia may be affected by neurotransmitters, chemokines and inflammatory cytokines. Furthermore, activation of the PI3K-AKT signaling pathway play a central role in the process of the apoptotic death of neural cells and causes neuroinflammation, which eventually leads to seizures or epilepsy. Mir-148a-3p inhibitor can inhibit the expression of mir-148a-3p, thereby increasing the expression of the SYNJ1 gene, making dephosphorylating PIP3 into PIP2, inhibiting PI3K-AKT signaling pathway, and reducing cell apoptosis. Conversely, upregulation of miR-148a-3p could enhance the PI3K-AKT mediated apoptosis pathway and neuroinflammation and then cause neuroinflammation, leading to seizures and epilepsy.

Our study detected the apoptosis rate of hippocampal neurons by Flow cytometry, which suggested that miR-148a-3p promoted the apoptosis of neurons. Previously, the PI3K/Akt signaling pathway was reported to modulate the survival response to neuronal apoptosis caused by oxidative stress following epileptic seizures^52^. Additionally, the neuronal inflammatory response induced by interleukin-1β stimulates the activation of the PI3K/Akt signaling pathway, which is involved in Pathophysiology of Seizures^53^. In order to further verify whether miR-148a-3p affects the PI3K/AKT signaling pathway in hippocampal neurons, western blot analysis demonstrated that levels of phosphorylation of PI3K and AKT are activated when transfected with the miR-148a-3p. Based on our findings, we propose a novel model wherein miR-148a-3p promote neuronal apoptosis via activation of PI3K-AKT signaling pathway by targeting SYNJ1 (Fig. 10).

In summary, through microarray analysis of miRNA expression profiles in the hippocampus of rats with recurrent FSs, a series of differentially expressed miRNAs and candidate target genes along with GO functional and KEGG pathway classifications were obtained. Our findings also demonstrate for the first time that the miR-148a-3p effectively promoted neuronal cell apoptosis via the activation of PI3K/AKT signaling pathway by controlling the expression of SYNJ1 in the neuronal cell, which provides new insights into a potential therapeutic target of miR-148a-3p in the modulation of neuron-mediated apoptosis and may be beneficial for the treatment of infants with FS brain injury. However, future research should study the effects of miR-148a-3p on specific epileptic phenotypes, such as abnormal neuronal circuits, spatial learning disruptions, and memory impairment. Additionally, considering the limiting factors, such as the blood–brain barrier, it is imperative to determine the clinical feasibility of targeting miR-148a-3p in the treatment of seizures or epilepsy.

## Materials and methods

### Ethics statement

All protocols complied with the recommendations in the Guide for the Care and Use of Animals for an Animal Biosafety Level 3 (ABSL-3) Laboratory and were approved by the Animal Ethics Committee of Wuhan University (Permit Number: SCXK 2016-0004). All surgery was performed under chloral hydrate anesthesia, and all efforts were made to minimize suffering.

### Establishment of the rat models of FS

Sprague–Dawley rats (aged 14 days) were obtained from an ABSL-3 laboratory. The rats were housed individually under a 12-h light–dark cycle with ad libitum access to food and water. Hyperthermia-induced seizures were produced using a hot-water bath^54^. The animals were placed in the temperature-controlled (39.5–42.5 °C) water bath and removed from the water immediately once seizures were induced. Seizures were induced once every day for 7 days.

After completion of the whole seizure induction process, the rats were anesthetized with 10% chloral hydrate (3 ml/kg). The brains were quickly removed and placed on ice, and the hippocampi were dissected, placed into liquid nitrogen and transferred into a − 80 °C low-temperature freezer for storage and later use.

### MiRNA array analysis

RNA was isolated by the TRIzol method (Invitrogen, Thermo Fisher, USA), and miRNA profiling was performed by Kang Chen Biosciences (Shanghai, China). Briefly, purified RNA was labeled using a miRCURY Hy3/Hy5 Power labeling kit and hybridized to a miRCURY LNA miRNA array (v.11.0) (Exiqon, Vedaek, Denmark). Scanning was performed with an Axon GenePix 4000B microarray scanner. GenePix Pro V6.0 was used to read the raw intensities of the images^55^. The results were subjected to unsupervised hierarchical clustering and tree analysis.

### MiRNA target prediction

All significant differentially expressed miRNAs were analyzed by bioinformatics algorithms. The potential target genes of these miRNAs were predicted using miRNA target prediction databases, including TargetScan (http://www.targetscan.org) and miRDB (http://www.mirdb.org).

### Gene Ontology and pathway enrichment analysis

Functional classification of all differentially expressed miRNA target genes was performed by DAVID v6.8 Database (https://david.ncifcrf.gov/) to determine the biological significance of the targets, and the accompanying p-values calculated by Fisher’s exact test indicated the functions that were overrepresented among the targets. The P-values produced by topGO denoted the significance of the enrichment of GO terms for the differentially expressed genes. A lower P-value indicated a more significant GO term (a P-value threshold of ≤ 0.05 is recommended). Moreover, a pathway analysis was performed using the Kyoto Encyclopedia of Genes and Genomes (KEGG) database to identify the enriched pathways of the targets^56–58^, and the P-value (significance) was calculated for each pathway using a hypergeometric distribution^59^. Furthermore, to perform the GO and pathway enrichment analyses of the miR-148a-3p target genes, we used the Metascape Database (http://metascape.org) with the custom analysis^60^.

### Integration of PPI network complex identification

We developed a miR-148a-3p target gene-encoded protein and PPI network using STRING (http://string-db.org)^61^. The PPI network was constructed using Cytoscape software (version 3.6.1) to analyze the interactions between predicted target gene-encoded proteins in FS^62^. The Node Analyzer was calculated using the Network Analyzer plug-in, which reveals the number of connections used to filter the PPI hub genes. The corresponding protein identified at the central node may be a core protein and key candidate gene with important physiological regulatory functions.

### Real-time quantitative PCR (qRT-PCR) analysis

Briefly, after hippocampal isolation and RNA extraction, cDNA was prepared using a RevertAid First Strand cDNA Synthesis Kit (Thermo Fisher, USA). qRT-PCR was performed using TaqMan MicroRNA Assays (Life Technologies, Carlsbad, CA, USA). All real-time reactions were performed in triplicate on an ABI PRISM 7000 sequence detection system (Ambion-Applied Biosystems, Foster City, CA, USA). The relative expression was calculated using the comparative cycle threshold (ct) method, and the relative expression ratio was determined by the formula 2^−△△ct^^63^.

### Terminal deoxynucleotidyl transferase-mediated dUTP nick-end labeling (TUNEL) assay

Apoptosis was determined using a TUNEL assay. The apoptotic index was calculated as the percentage of TUNEL-positive cells divided by the total number of cells. TUNEL in situ cell death detection kits were purchased from Roche^64^.

### Flow cytometry analysis

An annexin V-fluorescein isothiocyanate (FITC)/propidium iodide (PI) apoptosis detection kit (Cat No. 70-AP101-100; MultiSciences, Hangzhou, China) was used to evaluate the apoptotic rates of neuronal cells. After 48 h of transfection, neurons were treated with kainic acid (KA; 100 μM) for 24 h, and then the cells were collected, washed with PBS, and suspended with 5 µl of Annexin V-FITC and 5 µl of PI. The cells were then incubated for 30 min in the dark at room temperature. We used a flow cytometer (BD Biosciences) to analyze apoptosis and evaluate the apoptotic rate^65^.

### Dual-luciferase reporter assay

The gene SYNJ1 was predicted to be a target of miR-148a-3p by TargetScan (http://www.targetscan.org). To verify that SYNJ1 is a target gene of miR-148a-3p, the 3′UTR region of SYNJ1 was amplified, the PCR product was cloned into the downstream multiple cloning site of the PGL3 luciferase reporter vector (Promega Corporation, Madison, WI), and then site-directed mutagenesis was performed on the binding site between miR-148a-3p and the target gene, which was predicted by TargetScan. The Renilla luciferase reporter vector pRL-TK (TaKaRa Biotechnology Co. Ltd., Dalian, China) was chosen as the internal reference to adjust for differences in cell number and transfection efficiency. H19-7 hippocampal neuronal cells were separately cotransfected with a miR-148a-3p mimic or a mimic negative control (NC) and a luciferase reporter vector. Then, the luciferase activity was examined by the method provided by Promega^66^.

### Immunofluorescence staining and FISH

The procedure was performed as described in a previous study^67^. Briefly, segments of hippocampus were rapidly dissected from SD rat brains perfused with 4% paraformaldehyde, fixed with 4% PFA, and then cryoprotected in 30% sucrose. miR-148a-3p probes (5′-CAAAGTT CTGTAGTGCACTGA-3′) were synthesized (F03101, Gene Pharma). FISH was performed using the FISH kit (Guangzhou, Ribobio), and then FISH sections were incubated with MAP2 antibody (1:200, ab5392; Abcam), and GFAP antibody (1:100, #3670, Cell Signaling Technology), then with fluorescent-conjugated secondary antibody (1:200, 111-165-003, 115-165-003, Jackson) at 37 °C for 1 h. After the sections were rinsed in 0.01 M PBS, the slides were mounted on a mounting medium with DAPI and scanned via Ortho-Fluorescent Microscopy (Nikon).

### Western Blotting

Protein samples from the H19-7 hippocampus neurons were loaded to a 12% SDS-PAGE. PVDF membranes were used for the SDS-PAGE. The membrane was blotted for 2 h using 5% non-fat milk in the TBST solution.

Primary antibodies against PI3K, phosphorylate-PI3K (p-PI3K), Akt and phosphorylate Akt (p-Akt), and β-actin (1: 3000, Abcam, Cambridge, MA) were added for incubation at 4 °C overnight. Then, the membranes were incubated with a species-matched HRP-conjugated secondary antibody (Abcam) for 1 h at room temperature. Quantitative data of western blots were analysed using Gel-Pro3.0 software. The intensity of the tested protein bands was normalised to the internal reference.

### Statistical analysis

All data were analyzed using SPSS 21.0 integrated software (SPSS Inc. IBM, Chicago, IL). Measurement data are shown as the Mean ± standard deviation (SD). Comparisons between two groups and among multiple groups were performed via t test and one-way analysis of variance (ANOVA), respectively. A value of P < 0.05 was considered statistically significant.

## Supplementary Information

Supplementary Information.

Supplementary Information.

## Data Availability

All datasets generated during the study are available on request from the corresponding author.
