# Supplementary material for: MicroRNA expression profiling after recurrent febrile seizures in rat and emerging role of miR-148a-3p/SYNJ1 axis
Source: Sci Rep. 2021 Jan 13;11:1262. doi: 10.1038/s41598-020-79543-0 (PMC7806659; doi:10.1038/s41598-020-79543-0)
Supplement: Supplementary file 1 — Supplementary Information. [file 41598_2020_79543_MOESM1_ESM.pdf]

MicroRNA expression profiling after recurrent febrile seizures in rat and emerging role of miR-148a-3p/SYNJ1 axis

Jian Xu[1,2], Mingqiang Sun[2], Xiaodong Li[3], Lei Huang[4], Zhenzhong Gao[3*], Jian Gao[3*], Anmu Xie[1*]

1. Department of Neurology, The Affiliated Hospital of Qingdao University, Qingdao, 266000, China

2. Department of Clinical Lab, Maternal and Child Health Hospital of Weifang Medical University, 261011, China

3. Department of Pediatric, Maternal and Child Health Hospital of Weifang Medical University, 261011, China

4. Department of Cancer Blood Disease, Cincinnati Children's Hospital Medical Center, OH, 45229, USA

***These authors contributed equally to this article.**

Corresponding Authors:

Anmu Xie, Department of Neurology, The Affiliated Hospital of Qingdao University, Qingdao, 266000, China

E-mail:763050348@qq.com(AM.X);1395600477@qq.com(ZZ.G);

gaojian1650@126.com(J.G)

Supplementary Information

Table S1 Differential expression of miRNAs (JX)

Table S2 The primer sequence for RT-qPCR (JX)

Table S3 MiR-148a-3p target genes (JX)

Figure S1 Effects of different concentrations of KA on neuronal apoptosis in vitro. (JX)

Figure 9S Full length gels and blots

Figure S1Effects of different concentrations of KA on neuronal apoptosis in vitro

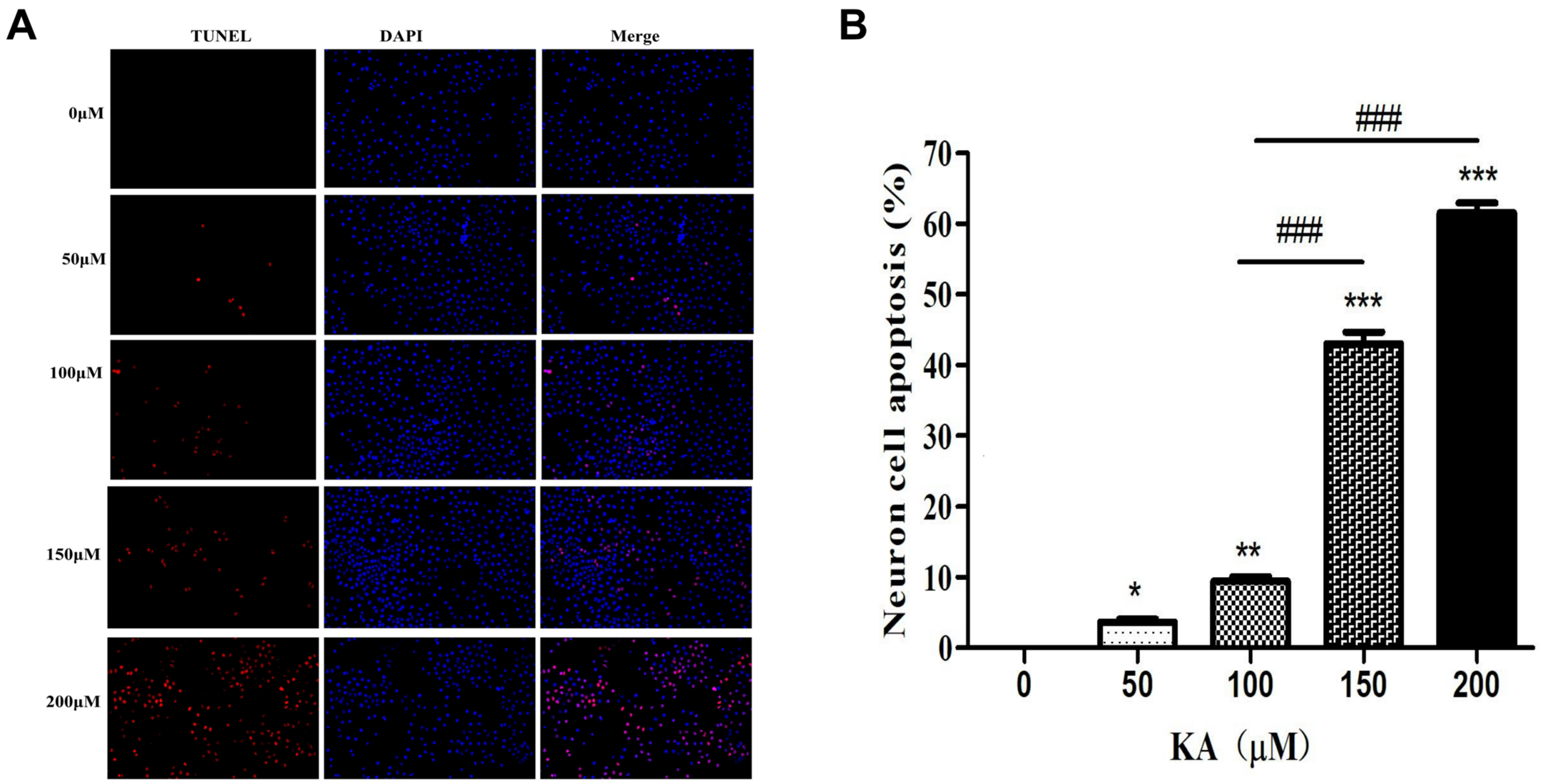

Fig 9S full length gels and blots

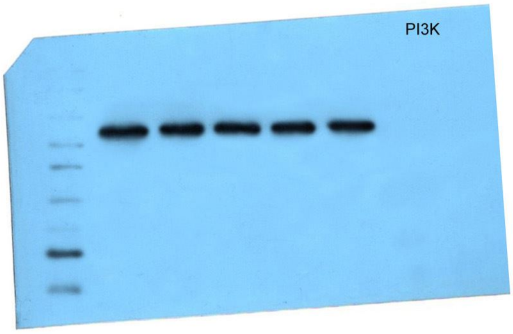

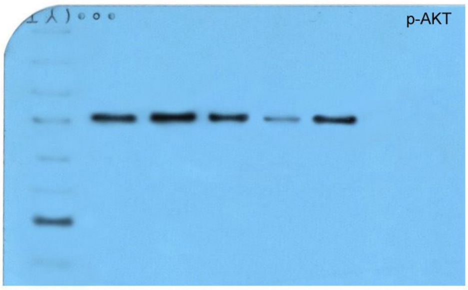

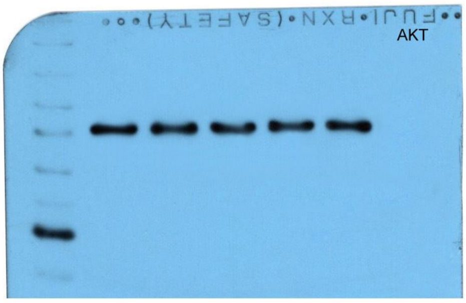

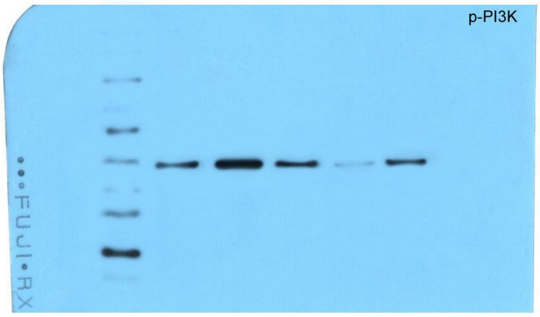

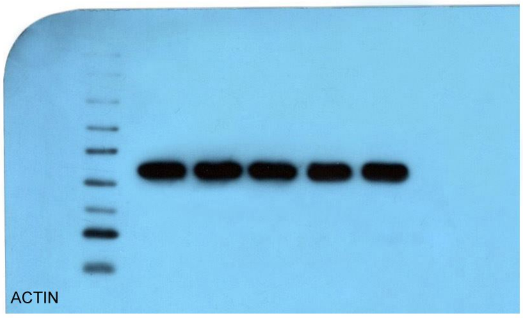

Table S1 Differential expression of miRNAs

ID	Name	Test vs Con_FC	Test vs Con_Pvalue	Test vs Con_FDR
42748	rno-miR-191a-3p	7.071481542	0.032384061	0.602001353
11077	rno-miR-363-3p	4.3788585	0.022551828	0.584553886
42811	rno-miR-542-5p	4.311581158	0.00117141	0.205430325
148576	rno-miR-92a-2-5p	4.078159806	0.005040252	0.371521114
14313	rno-miR-499-5p	3.534540198	0.013588283	0.480306902
17312	rno-miR-592	2.407278178	0.01693553	0.536444344
148147	rno-miR-181b-1-3p	2.323103668	0.007404809	0.407713371
10955	rno-miR-148a-3p	2.069458384	0.04870889	0.653096436
4700	rno-miR-140-5p	1.90896304	0.003447325	0.317769452
46223	rno-miR-1306-3p	1.906452639	0.002920967	0.295227729
46859	rno-miR-135a-3p	1.892291133	0.002297872	0.283484803
42866	rno-miR-451-5p	1.860332571	0.006109933	0.407713371
13140	rno-miR-138-5p	1.780263803	0.011860788	0.480306902
11074	rno-miR-34c-5p	1.740133325	0.041209944	0.627792797
46483	rno-miR-27a-3p	1.723976658	0.043543536	0.63392998
10947	rno-miR-142-3p	1.678026702	0.00060727	0.158160198
11013	rno-miR-181a-1-3p	1.646201184	0.030318381	0.602001353
17853	rno-miR-30d-3p	1.582516121	0.02461559	0.598156347
147506	rno-miR-21-5p	1.565314452	0.044353463	0.63392998
11018	rno-miR-218a-5p	1.559778541	0.025040523	0.598156347
14301	rno-miR-361-5p	1.518949578	0.043535445	0.63392998
148078	rno-miR-3577	0.658493957	0.01319288	0.480306902
11208	rno-miR-207	0.593832259	0.027433407	0.602001353
148614	rno-miR-7a-2-3p	0.553817104	0.040946497	0.627792797
148229	rno-miR-3557-5p	0.494488002	0.013728909	0.480306902
28019	rno-miR-10a-3p	0.490078056	0.030509321	0.602001353
148476	rno-miR-3552	0.477904304	0.00302281	0.295227729
10936	rno-miR-130b-3p	0.392676821	0.0262335	0.602001353
148511	rno-miR-3595	0.392543353	0.031171689	0.602001353
148455	rno-miR-741-3p	0.231621332	0.037548598	0.62421216
148201	rno-miR-365-5p	0.193823827	0.006546783	0.407713371

Table S2 The primer sequence for RT-qPCR.

miRNA name	Primer sequence
U6	F:5’-GCTTCGGCAGCACATATACTAAAAT-3’<br>R:5’-CGCTTCACGAATTTGCGTGTCAT-3’
rno-miR-138-5p	F:5’- GGGGCTGGTGTTGTGAATC-3’<br>R:5’-CAGTGCGTGTCGTGGAGT-3’
rno-miR-451-5p	F:5’ -GGGGGAAACCGTTACCATTAC-3’<br>R:5’-CAGTGCGTGTCGTGGAGT-3’
rno-miR-499-5p	F:5’- GGGGGTTAAGACTTGCAGTG-3’<br>R:5’-CAGTGCGTGTCGTGGAGT-3’
rno-miR-148a-3p	F:5’ -GGGTCAGTGCACTACAGA- 3’<br>R:5’-CAGTGCGTGTCGTGGAGT-3’
rno-miR-542-5p	F:5’- GGCTCGGGGATCATCAT- 3’<br>R:5’-CAGTGCGTGTCGTGGAGT-3’
rno-miR-130b-3p	F:5’- CCAGTTACCGCTTCCG- 3’<br>R:5’-CAGTGCGTGTCGTGGAGT-3’
rno-miR-207	F:5’- GGCTTCTCCTGGCTCTCCT-3’<br>R:5’-CAGTGCGTGTCGTGGAGT-3’
rno-miR-3577	F:5’ -TTTGCCCACCCTTTACCC- 3’<br>R:5’-CAGTGCGTGTCGTGGAGT-3’
rno-miR-7a-2-3p	F:5’ -GGAAGGTGGGGATTAGTGC-3’<br>R:5’-CAGTGCGTGTCGTGGAGT-3’

TableS3 MiR-148a-3p target genes

Gene ID	Gene Symbol	Description
25270	Abcd3	ATP binding cassette subfamily D member 3
300474	Adamts15	ADAM metallopeptidase with thrombospondin type 1 motif, 15
81636	Adcy2	adenylate cyclase 2
363266	Agfg1	ArfGAP with FG repeats 1
313594	Ago1	argonaute RISC component 1
298533	Ago4	argonaute RISC component 4
362617	Ahdc1	AT hook, DNA binding motif, containing 1
85265	Ajuba	ajuba LIM protein
79559	Alcam	activated leukocyte cell adhesion molecule
171494	Ap1g1	adaptor related protein complex 1 subunit gamma 1
312915	Arfgef1	ADP ribosylation factor guanine nucleotide exchange factor 1
60382	Arfip1	ADP-ribosylation factor interacting protein 1
307178	Arhgap21	Rho GTPase activating protein 21
307398	Arhgef37	Rho guanine nucleotide exchange factor 37
293551	Arl6ip1	ADP-ribosylation factor like GTPase 6 interacting protein 1
500282	Arl8b	ADP-ribosylation factor like GTPase 8B
305831	Atg14	autophagy related 14
29693	Atp2a2	ATPase sarcoplasmic/endoplasmic reticulum Ca2+ transporting 2
24941	Atp7a	ATPase copper transporting alpha
25049	Atxn1	ataxin 1
313125	Bach2	BTB domain and CNC homolog 2
64547	Bcl2l11	BCL2 like 11
361832	Bicc1	BicC family RNA binding protein 1
25667	Bmp3	bone morphogenetic protein 3
679713	Brpf1	bromodomain and PHD finger containing, 1
368042	Btaf1	B-TFIID TATA-box binding protein associated factor 1
65044	C1galt1	glycoprotein-N-acetylgalactosamine 3-beta-galactosyltransferase, 1
360970	Cabp7	calcium binding protein 7
64202	Calr	calreticulin
296580	Camsap1	calmodulin regulated spectrin-associated protein 1
117152	Cand1	cullin-associated and neddylation-dissociated 1
29144	Canx	calnexin
498678	Ccdc71	coiled-coil domain containing 71
117524	Ccnf	cyclin F
310806	Cdc14a	cell division cycle 14A
309804	Cdk19	cyclin-dependent kinase 19
308215	Chd1	chromodomain helicase DNA binding protein 1
140583	Chek1	checkpoint kinase 1
309361	Chuk	component of inhibitor of nuclear factor kappa B kinase complex
84360	Clcn3	chloride voltage-gated channel 3
60447	Clock	clock circadian regulator
25248	Cnr1	cannabinoid receptor 1
116658	Cntn4	contactin 4
25412	Col2a1	collagen type II alpha 1 chain
290905	Col4a1	collagen type IV alpha 1 chain

78965	Csf1	colony stimulating factor 1
310760	Cttnbp2nl	CTTNBP2 N-terminal like
171015	Cyb5r4	cytochrome b5 reductase 4
360928	Dcun1d4	defective in cullin neddylation 1 domain containing 4
500988	Ddx6	DEAD-box helicase 6
287954	Dgcr8	DGCR8 microprocessor complex subunit
291760	Dsc2	desmocollin 2
140734	Dynll2	dynein light chain LC8-type 2
286973	Elavl2	ELAV like RNA binding protein 2
432358	Elavl4	ELAV like RNA binding protein 4
366142	Elf5	E74 like ETS transcription factor 5
499380	Emx2	empty spiracles homeobox 2
60443	Epn2	epsin 2
293939	Erlin1	ER lipid raft associated 1
313729	Errfi1	ERBB receptor feedback inhibitor 1
360896	Esrrg	estrogen-related receptor gamma
308999	Fbxl19	F-box and leucine-rich repeat protein 19
314157	Fbxo33	F-box protein 33
309129	Fcho2	FCH domain only 2
24948	Fmr1	fragile X mental retardation 1
303753	Foxk2	forkhead box K2
25112	Gadd45a	growth arrest and DNA-damage-inducible, alpha
29423	Gap43	growth associated protein 43
50564	Gjd2	gap junction protein, delta 2
362776	Glrx5	glutaredoxin 5
81661	Gmfb	glia maturation factor, beta
306439	Gpm6a	glycoprotein m6a
79220	Grid2	glutamate ionotropic receptor delta type subunit 2
361580	Gtf2h1	general transcription factor IIH subunit 1
117140	Hivep1	human immunodeficiency virus type I enhancer binding protein 1
313557	Hivep3	human immunodeficiency virus type I enhancer binding protein 3
315689	Hmg20a	high mobility group 20A
29546	Homer1	homer scaffold protein 1
290744	Ing2	inhibitor of growth family, member 2
25196	Inhbb	inhibin subunit beta B
296084	Ino80	INO80 complex ATPase subunit
315744	Itga11	integrin subunit alpha 11
315346	Itga5	integrin subunit alpha 5
307916	Jph3	junctophilin 3
303470	Kat7	lysine acetyltransferase 7
304495	Kdm2b	lysine demethylase 2B
114505	Klf4	Kruppel like factor 4
58954	Klf6	Kruppel-like factor 6
360983	Lgalsl	galectin-like
25055	Lipa	lipase A, lysosomal acid type
100909856	LOC10090985	metal regulatory transcription factor 1-like
83469	Lrp4	LDL receptor related protein 4

59107	Ltbp1	latent transforming growth factor beta binding protein 1
315093	Maf1	MAF1 homolog, negative regulator of RNA polymerase III
500690	Map3k9	mitogen-activated protein kinase kinase kinase 9
313997	Mboat2	membrane bound O-acyltransferase domain containing 2
362325	Mdfic	MyoD family inhibitor domain containing
29279	Meox2	mesenchyme homeobox 2
313418	Mier1	MIER1 transcriptional regulator
361285	Mllt10	MLLT10, histone lysine methyltransferase DOT1L cofactor
303439	Mmd	monocyte to macrophage differentiation-associated
291848	Mmp15	matrix metallopeptidase 15
287521	Mnt	MAX network transcriptional repressor
317312	Mospd1	motile sperm domain containing 1
25482	Mras	muscle RAS oncogene homolog
309255	Mtmr10	myotubularin related protein 10
312634	Mtmr14	myotubularin related protein 14
361500	Nat14	N-acetyltransferase 14
301009	Nckipsd	NCK interacting protein with SH3 domain
313929	Ncoa1	nuclear receptor coactivator 1
54299	Ncor1	nuclear receptor co-repressor 1
303248	Neurl4	neuralized E3 ubiquitin protein ligase 4
25495	Nog	noggin
307553	Nol4	nucleolar protein 4
298992	Nova1	NOVA alternative splicing regulator 1
311671	Npepl1	aminopeptidase-like 1
56064	Nptn	neuroplastin
266777	Nptx1	neuronal pentraxin 1
361128	Nr2c2ap	nuclear receptor 2C2-associated protein
499745	Nrarp	Notch-regulated ankyrin repeat protein
361736	Patl1	PAT1 homolog 1, processing body mRNA decay factor
29468	Pdia3	protein disulfide isomerase family A, member 3
364052	Pea15	proliferation and apoptosis adaptor protein 15
363210	Phf3	PHD finger protein 3
116645	Plaa	phospholipase A2, activating protein
314262	Plekhh1	pleckstrin homology, MyTH4 and FERM domain containing H1
29588	Pou3f2	POU class 3 homeobox 2
362876	Ppfia2	PTPRF interacting protein alpha 2
25594	Ppp1cb	protein phosphatase 1 catalytic subunit beta
65045	Ppp1r10	protein phosphatase 1, regulatory subunit 10
84686	Ppp1r9b	protein phosphatase 1, regulatory subunit 9B
312563	Prickle2	prickle planar cell polarity protein 2
65248	Prkaa1	protein kinase AMP-activated catalytic subunit alpha 1
373545	Prkag2	protein kinase AMP-activated non-catalytic subunit gamma 2
362809	Ptges3	prostaglandin E synthase 3
360571	Rab34	RAB34, member RAS oncogene family
294804	Rai14	retinoic acid induced 14
84014	Ralbp1	ralA binding protein 1
690139	Rbm24	RNA binding motif protein 24

315804	Rfx7	regulatory factor X, 7
308739	Rgma	repulsive guidance molecule BMP co-receptor a
171501	Rnf38	ring finger protein 38
84409	Robo2	roundabout guidance receptor 2
171123	Rph3al	rabphilin 3A-like (without C2 domains)
314384	Rps6ka5	ribosomal protein S6 kinase A5
29733	S1pr1	sphingosine-1-phosphate receptor 1
24617	Serpine1	serpin family E member 1
502988	Sesn2	sestrin 2
59329	Sik1	salt-inducible kinase 1
303745	Sirt7	sirtuin 7
287280	Skp1	S-phase kinase-associated protein 1
365841	Slc25a44	solute carrier family 25, member 44
24778	Slc2a1	solute carrier family 2 member 1
29357	Smad2	SMAD family member 2
363469	Sms	spermine synthase
303614	Smurf2	SMAD specific E3 ubiquitin protein ligase 2
29140	Snn	stannin
260323	Snx27	sorting nexin family member 27
85384	Sos2	SOS Ras/Rho guanine nucleotide exchange factor 2
502603	Srsf11	serine and arginine rich splicing factor 11
361233	Ssr1	signal sequence receptor subunit 1
25547	St8sia3	ST8 alpha-N-acetyl-neuraminide alpha-2,8-sialyltransferase 3
498798	Stam	signal transducing adaptor molecule
498130	Stard13	StAR-related lipid transfer domain containing 13
500972	Stt3a	STT3 oligosaccharyltransferase complex catalytic subunit A
81802	Stx3	syntaxin 3
85238	Synj1	synaptojanin 1
500575	Szrd1	SUZ RNA binding domain containing 1
24827	Tgfa	transforming growth factor alpha
309126	Tnpo1	transportin 1
308971	Tnrc6a	trinucleotide repeat containing adaptor 6A
171086	Trak2	trafficking kinesin protein 2
361831	Ube2d1	ubiquitin-conjugating enzyme E2D 1
81920	Ube2d3	ubiquitin-conjugating enzyme E2D 3
687633	Ube3b	ubiquitin protein ligase E3B
25708	Ucp3	uncoupling protein 3
303394	Usp32	ubiquitin specific peptidase 32
310960	Usp33	ubiquitin specific peptidase 33
308896	Usp47	ubiquitin specific peptidase 47
314453	Wdr20	WD repeat domain 20
24881	Wnt1	Wnt family member 1
290280	Xpo4	exportin 4
293491	Ypel3	yippee-like 3
56011	Ywhab	tryptophan 5-monooxygenase activation protein, beta
315211	Zbed4	zinc finger, BED-type containing 4
314265	Zfyve26	zinc finger FYVE-type containing 26
