# Supplementary material for: MicroRNA expression profiling after recurrent febrile seizures in rat and emerging role of miR-148a-3p/SYNJ1 axis
Source: Sci Rep. 2021 Jan 13;11:1262. doi: 10.1038/s41598-020-79543-0 (PMC7806659; doi:10.1038/s41598-020-79543-0)
Supplement: Supplementary file 2 — Supplementary Information. [file 41598_2020_79543_MOESM2_ESM.docx]

**Figure S1 Effects of different concentrations of KA on neuronal apoptosis in vitro.**

(A) Effects of different concentrations of KA on the apoptosis rate of hippocampal neuronal cells by TUNEL staining. (B) Bar chart showing the results of the statistical analysis of neuronal apoptosis. The data were analyzed by t test or one-way ANOVA; the pairwise comparison after ANOVA was analyzed by the LSD-t test; *, P < 0.05 vs. the control group; ###, P < 0.001, 100 µM group VS 150 µM group and 200 µM group.
